# Motor Abnormalities in Attention-Deficit/Hyperactivity Disorder and Autism Spectrum Disorder Are Associated With Regional Grey Matter Volumes

**DOI:** 10.3389/fneur.2021.666980

**Published:** 2021-05-04

**Authors:** Ariadna Albajara Sáenz, Thomas Villemonteix, Peter Van Schuerbeek, Simon Baijot, Mathilde Septier, Pierre Defresne, Véronique Delvenne, Gianfranco Passeri, Hubert Raeymaekers, Laurent Victoor, Eric Willaye, Philippe Peigneux, Nicolas Deconinck, Isabelle Massat

**Affiliations:** ^1^Neuropsychology and Functional Neuroimaging Research Group (UR2NF) at the Centre for Research in Cognition and Neurosciences, Université Libre de Bruxelles, Brussels, Belgium; ^2^Paris 8 Vincennes - St Denis University, Laboratoire de Psychopathologie et Neuropsychologie, Saint Denis, France; ^3^Department of Radiology, Universitair Ziekenhuis Brussel, Brussels, Belgium; ^4^Hôpital Universitaire des Enfants Reine Fabiola (HUDERF), Université Libre de Bruxelles, Brussels, Belgium; ^5^Hôpital Universitaire Robert Debré, Paris, France; ^6^Institut de Psychiatrie et de Neurosciences de Paris Inserm U894 Team 1, Paris, France; ^7^Fondation SUSA (Service Universitaire Spéécialisé pour personnes avec Autisme), Université de Mons, Mons, Belgium; ^8^PsyPluriel, Centre Européen de Psychologie Médicale, Brussels, Belgium; ^9^Laboratory of Experimental Neurology, Université Libre de Bruxelles, Brussels, Belgium; ^10^National Fund of Scientific Research, Brussels, Belgium; ^11^Department of Neurology, Erasme Hospital, Brussels, Belgium

**Keywords:** attention-deficit/hyperactivity disorder, autism spectrum disorder, voxel-based morphometry, DCDQ, motor performance

## Abstract

Attention-Deficit/Hyperactivity Disorder (ADHD) and Autism Spectrum Disorder (ASD) are associated with motor impairments, with some children holding a comorbid diagnosis of Developmental Coordination Disorder (DCD). However, DCD is underdiagnosed in these populations and the volume abnormalities that contribute to explaining these motor impairments are poorly understood. In this study, motor abilities as measured by the Developmental Coordination Disorder Questionnaire (DCDQ) were compared between children with ADHD, children with ASD and typically developing (TD) children, aged 8–12 years old. Additionally, the association between the DCDQ scores (general coordination, fine motor/handwriting, control during movement, total) and regional volume abnormalities were explored in 6 regions of interest (pre-central gyrus, post-central gyrus, inferior parietal cortex, superior frontal gyrus, middle frontal gyrus, medial frontal gyrus), within each group and across all participants. Children with ASD and children with ADHD showed impaired motor abilities in all the DCDQ-derived scores compared to TD children. Additionally, most children with ASD or ADHD had an indication or suspicion of DCD. Within the ASD group, coordination abilities were associated with the volume of the right medial frontal gyrus, and within the ADHD group, the total DCDQ score was associated with the volume of the right superior frontal gyrus. This study underlines the importance of routinely checking motor abilities in populations with ASD or ADHD in clinical practise and contributes to the understanding of structural abnormalities subtending motor impairments in these disorders.

## Introduction

Attention-Deficit/Hyperactivity Disorder (ADHD) is defined by impairing levels of inattention, and/or hyperactivity-impulsivity (1). Autism Spectrum Disorder (ASD) is characterised by impaired social communication and interaction, and restricted, repetitive patterns of behaviour, interests or activities (1). Although ASD and ADHD are characterised by distinct diagnostic criteria, these two disorders often co-occur (2, 3). In clinical practise, some symptoms associated with both ASD and ADHD can make it difficult to differentiate between these disorders (4). Abnormal motor activity is frequent in both ADHD and ASD (1). In ASD, atypical motor functioning goes beyond repetitive movements, affecting a wide range of motor skills such as motor coordination and postural stability, leading some authors to propose motor deficits as a potential core feature of ASD (5). Importantly, motor delays during early childhood are one of the earliest markers of ASD (6). In ADHD, abnormal motor activity is characterised by fidgeting and restlessness, and children with ADHD can experience difficulties with gross and fine motor skills (1, 7, 8). Additionally, ASD and ADHD are often accompanied by the Developmental Coordination Disorder (DCD), a motor disorder characterised by deficits in the acquisition and execution of coordinated motor skills (1, 9–12). These difficulties are manifested by clumsiness and slowness or inaccuracy of performance of motor skills and interfere with activities of daily living (1). However, the DCD seems to be underdiagnosed in children with ASD or ADHD (10, 12).

The Developmental Coordination Disorder Questionnaire (DCDQ) is considered a valid screening tool for identifying children with possible DCD (13, 14) and has been used in populations with ASD and ADHD (10, 12, 15, 16). In a sample of 11,814 children with ASD, Bhat (12) found that only 15.1% of the children had a formal comorbid diagnosis of DCD, while 86.9% of the participants were identified as at risk for DCD based on the DCDQ, suggesting that motor impairments in ASD are under-recognised. Although comorbidity rates vary between studies, it is estimated that ~30–55% of children with ADHD manifest DCD (9, 10). In a group of 486 children with ADHD, Fliers et al. (10) found that around 30% of the girls and 33% of the boys had DCDQ scores below the 10th percentile cut-off, indicating the presence of motor coordination difficulties. These studies show that a high proportion of individuals with ASD and ADHD show impairments at the DCDQ. However, it is yet unknown if the DCDQ could assist in differentiating between ASD and ADHD.

At the structural level, voxel-based morphometry (VBM) studies have evidenced volumetric abnormalities in populations with ADHD and/or ASD in regions typically associated with motor performance, including the pre-central and post-central gyri and the inferior parietal cortex (17–19). A recent study by Mahajan et al. (17) compared the volume of the pre-central gyrus, the post-central gyrus and the inferior parietal cortex between a group of children with ASD, a group of children with comorbid ASD and ADHD and a group of typically developing (TD) children. Children with ASD showed abnormal increases in grey matter volume (GMV) in these three regions. Additionally, increased GMV in the left post-central gyrus and in the right pre-central gyrus was specific to children with ASD without ADHD. On the other hand, volumetric abnormalities associated with DCD have only been investigated in one recent study: comparing 22 children with DCD and 22 TD children, Reynolds et al. (20) found that children with DCD had significantly decreased GMV in the right superior, middle and medial frontal gyri compared to the TD group.

Overall, there is limited research on the association between motor performance measures and brain volume in populations with ASD and/or ADHD (17). Mahajan et al. (17) found that impaired praxis and manual dexterity measures were associated with grey matter volume in the post-central gyrus in children with ASD and ADHD without a formal diagnosis of DCD. Manual dexterity performance was also associated with the volume of the post-central gyrus in children with ASD without ADHD. The exploration of the association between motor ability measures and grey matter volume might help identifying the brain abnormalities contributing to the motor impairments frequently encountered in these disorders. Therefore, in this study, we compared motor coordination abilities between children with ADHD, children with ASD and TD children, aged 8–12 years old, using the Developmental Coordination Disorder Questionnaire-French Canadian (DCDQ-FC). Additionally, we investigated the associations between variations in DCDQ-FC scores and the volume of 6 regions of interest (the pre-central gyrus, the post-central gyrus, the inferior parietal cortex, the superior frontal gyrus, the middle frontal gyrus and the medial frontal gyrus) within each group and across participants, in order to investigate the full spectrum of impairments while accounting for heterogeneity. Based on previous literature, we hypothesised (1) that children with ADHD and ASD would exhibit increased levels of motor impairments as measured by the DCDQ-FC, and (2) that volumetric variations of the regions of interest studied will be associated with impairments in motor functions.

## Method

### Participants

The final sample of this study consisted of 22 children with ADHD, 16 children with ASD and 17 TD children aged 8–12 years old. The comparison of regional and total brain volumes between these groups of children is published elsewhere (21). Children with ADHD were recruited at the Erasme Hospital Department of Neurology and PsyPluriel-Pastur in Belgium. Diagnoses were assessed by trained child psychiatrists according to the DSM-5 criteria (1). All children with ADHD met the full Kiddie-Sads-Present and Lifetime Version (22) criteria for ADHD (combined-type), were medication-naïve and did not present any psychiatric comorbidity. Children with ASD were recruited in autism reference centres at the Queen Fabiola Children's University Hospital and the SUSA Foundation in Belgium. Diagnoses were assessed by trained child psychiatrists according to the DSM-5 criteria (1), using the Autism Diagnostic Interview-Revised (23), the Autism Diagnostic Observation Schedule (24) and/or the Childhood Autism Rating Scale (25). In the ASD group, eleven participants had a comorbid ADHD diagnosis, but did not present any other comorbidity. Additionally, one participant had a history of methylphenidate and risperidone intake, one was currently taking psychostimulant medication but underwent a 24-h washout period before the MRI scanning, and another participant was taking aripiprazole at the time of the scanning. Finally, TD children participated upon announcement or personal query. The parents of all participants filled the ADHD Rating Scale-IV- ADHD RS-IV (26) and the Developmental Coordination Disorder Questionnaire- French Canadian - DCDQ-FC [(14); see below].

Participants were excluded if they had a history of prematurity, a neurological disorder, a genetic disease, a disabling somatic pathology, suffered from complications during labour with neonatal care unit hospitalisation, were left-handed, presented any contraindications to MRI or had a General Ability Index lower than 70 on the Wechsler Intelligence Scale for Children−4th Edition (27, 28). Seventy-one children were initially enrolled in this study and 15 participants were excluded a posteriori: one after the fortuitous discovery of a brain anomaly during the MRI scan, 13 participants because of poor image quality and two participants because their DCDQ-FC results were missing. Participants received 50 euros to cover transportation expenses and written informed consent was obtained from their parents. This study was approved by the Ethics Committee of the ULB-Erasme University Hospital in Belgium (P2014/120; CCB: B406201420437).

### The Developmental Coordination Disorder Questionnaire-French Canadian DCDQ-FC

The DCDQ-FC (13, 14) is a brief parental questionnaire designed as a screening tool for identifying children aged 5–15 years old with possible DCD. Parents are asked to compare their child's coordination and motor abilities to that of children of the same age on a 5-point Likert scale (14). The questionnaire comprises 15 questions classified into three categories: general coordination, fine motor/handwriting and control during movement (14). The total score interpretation allows indicating whether the child's score is an “Indication of, or Suspect for, DCD,” or “Probably not DCD.”

### Brain Imaging

Anatomical image acquisition was conducted using a Discovery MR750w 3.0T scanner (GE Medical Systems, Milwaukee, Wisconsin, USA) at UZ Brussel [for details, see (21)]. Anatomical images were obtained using a T1-weigthed sagittal 3D TFE (turbo field echo) sequence: repetition time = 8.644 ms, echo time = 3.244 ms, inversion time = 450 ms, flip angle = 12°, field of view = 240 × 240 mm^2^, matrix size = 256 × 256 × 128 and voxel size = 0.94 × 0.94 × 1.2 mm^3^.

Imaging data were analysed using the Computational Anatomy Toolbox (CAT12; http://www.neuro.uni-jena.de/cat12/CAT12-Manual.pdf), implemented in SPM12 (http://www.fil.ion.ucl.ac.uk/spm/) running on MATLAB (R2019a, The MathWorks, Inc., Natick, MA). Image pre-processing and quality control is described elsewhere (21). Thirteen scans were excluded due to poor image quality. Images were excluded if their weighted average Image Quality Rating (IQR) was lower than 80%, corresponding to a “good” image quality (21). Image Quality Rating did not significantly differ between the ASD, ADHD, and TD children groups [*F*_(2, 52)_ = 0.32, *p* = 0.72].

Following pre-processing and data quality control, individual subject smoothed grey matter volumes were entered into a second level analysis to estimate the correlation between the different DCDQ-FC scores and grey matter volume in the ROIs using a one-sample *t*-test. Correlation coefficients were computed within each group (ADHD, ASD, TD) and across all participants. Global scaling was used with total intracranial volume (TIV) and age was entered as a covariate. All analyses were conducted without IQ as a covariate (21, 29). Masks were created for 12a priori ROIs (right and left pre-central gyrus, right and left post-central gyrus, right and left inferior parietal cortex, right and left superior frontal gyrus, right and left middle frontal gyrus and right and left medial frontal gyrus), defined using the Automated Anatomical Labelling atlas or the Talairach Daemon atlas implemented in the WFU PickAtlas toolbox (30). Within-mask area inferences were made using a small volume correction (SVC), and the *p*-value was Bonferroni-adjusted for the number of regions examined: *p*^SVC−FWE^ = 0.05/12 = 0.004. The initial voxel threshold was set to 0.001 at the whole brain level uncorrected and results were considered significant at *p*^SVC−FWE^ = 0.004. For the sake of completeness, findings that did not survive correction for multiple comparison are also reported (*p*^SVC−FWE^ = 0.05).

## Results

### Participant Characteristics

Sex was not significantly related to diagnosis and groups did not differ on age (see Table 1). IQ was significantly higher in the TD group compared to either the ASD (*p* = 0.011) or the ADHD (*p* = 0.004) groups. Groups differed significantly on all the ADHD RS-IV scores (all *p*s < 0.001). Total, Inattention and Hyperactivity scores were significantly higher in both the ADHD and the ASD groups compared to the TD group. These scores were not significantly different between the ADHD and the ASD groups. Additional demographic data analyses fractionating the ASD group in terms of the presence or absence of comorbid ADHD are reported in Supplementary Table 1.

**Table 1:** Demographic data.

	**ADHD (*****n*** **=** **22)**		**ASD (*****n*** **=** **16)**		**TD (*****n*** **=** **17)**		**Between-group difference**			***Post-hoc***
	**M/F**		**M/F**		**M/F**		**χ^**2**^**	**df**	***p***	
Sex	16/6		14/2		12/5		1.57	2	0.46	ns
	**M**	**SD**	**M**	**SD**	**M**	**SD**	**H**	**df**	***p***	
Age (months)	122	18.51	127	16.29	126.12	19.48	0.97	2	0.62	ns
IQ	100.23	13.74	99.62	17.68	118.59	14.93	12.52	2	0.002	ADHD, ASD < TD
**ADHD RS-IV**											
Total	33.91	9.96	22.44	9.08	7.35	4.8	35.87	2	<0.001	ADHD, ASD > TD
Inattention	19.27	4.38	13.62	7.14	4.18	2.67	31.06	2	<0.001	ADHD, ASD > TD
Hyperactivity-impulsivity	14.64	7.32	8.81	3.95	3.18	3.4	25.36	2	<0.001	ADHD, ASD > TD

*ADHD, Attention-Deficit/Hyperactivity Disorder; ASD, Autism Spectrum Disorder; TD, typically developing; M/F, Male/Female; χ^2^, Pearson's Chi-squared test; M, mean; SD, Standard deviation; H, test statistic for the Kruskal-Wallis test; df, degrees of freedom; p, p-value; ns, not significant; ADHD RS-IV, ADHD Rating Scale-IV*.

### Behavioural Data (DCDQ-FC)

Groups differed significantly on all the DCDQ-FC scores (all *p*s < 0.001). “Control During Movement,” “Fine Motor/Handwriting,” “General Coordination” and Total scores were significantly lower in both the ADHD and the ASD groups compared to the TD group (Table 2). These scores were not significantly different between the ADHD and the ASD groups. According to DCDQ-FC scores interpretation, 72.73% of the participants with ADHD had an indication of, or suspicion for DCD; 81.25% of the participants with ASD had an indication of, or suspicion for DCD; and none of the TD children had an indication of, or suspicion for DCD. Additional behavioural analyses fractionating the ASD group in terms of the presence or absence of comorbid ADHD are reported in the Supplementary Table 2.

**Table 2:** Motor coordination (DCDQ-FC) scores.

	**ADHD (*****n*** **=** **22)**		**ASD (*****n*** **=** **16)**		**TD (*****n*** **=** **17)**		**Between-groups difference**			***Post-hoc***
	**M**	**SD**	**M**	**SD**	**M**	**SD**	**H**	**df**	***p***	
Total	51.18	12.31	42.5	13.14	67.76	4.56	27.24	2	<0.001	ADHD, ASD < TD
Control during movement	22.09	6.11	17.19	5.64	27.18	2.3	21.02	2	<0.001	ADHD, ASD < TD
Fine motor/handwriting	12	4.4	11.94	4.61	17.82	2.32	18.09	2	<0.001	ADHD, ASD < TD
General coordination	16.64	4.58	13.19	5.33	22.76	1.71	27.31	2	<0.001	ADHD, ASD < TD

*ADHD, Attention-Deficit/Hyperactivity Disorder; ASD, Autism Spectrum Disorder; TD, typically developing; M/F, Male/Female; χ^2^, Pearson's Chi-squared test; M, mean; SD, Standard deviation; H, test statistic for the Kruskal-Wallis test; df, degrees of freedom; p, p-value*.

### Brain Imaging Data (Correlation Analyses)

**Figure 1:**
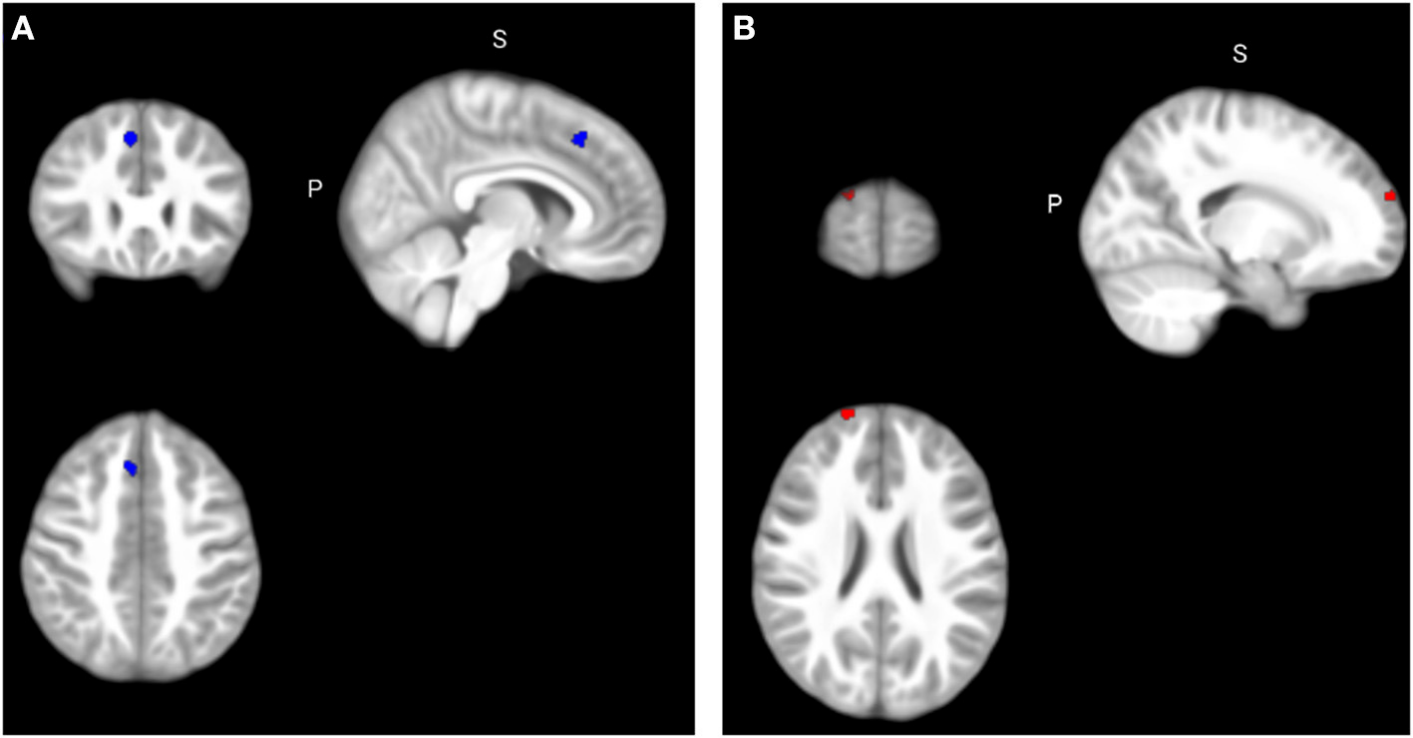
Correlation between DCDQ-FC scores and regional grey matter volumes. **(A)** Significant negative correlation between the General Coordination score and GMV in the right medial frontal gyrus within the ASD group (x = 6, y = 27, z = 46) at *p*^SVC−FWE^ < 0.004; **(B)** Significant negative correlation between the Total score and GMV in the right superior frontal gyrus within the ADHD group (x = 18, y = 68, z = 24) at *p*^SVC−FWE^ < 0.05. FWE, Family-Wise Error.

Correlation analyses between DCDQ-FC scores and regional grey matter volume in *a priori* ROIs evidenced a negative correlation between the DCDQ-FC General Coordination score and GMV in the right medial frontal gyrus within the ASD group, and a negative correlation between the DCDQ-FC General Coordination score and GMV in the left pre-central gyrus within the TD group (Bonferroni corrected threshold *p*^SVC−FWE^ < 0.004; Figure 1).

Additional analyses conducted for the sake of completeness (Table 3) at a lower threshold *p*^SVC−FWE^ < 0.05 revealed a negative correlation between the DCDQ-FC Total score and GMV in the right superior frontal gyrus within the ADHD group, and a negative correlation between the DCDQ-FC Total score and GMV in the right post-central gyrus within the ASD group. Within the TD group, ROI analyses yielded a negative correlation between the DCDQ-FC General Coordination score and GMV in the left post-central gyrus and a positive correlation between the DCDQ-FC Fine Motor/Handwriting score and GMV in the right medial frontal gyrus. Finally, across all participants, ROI analyses evidenced a negative correlation between the DCDQ-FC Control During Movement score and GMV in the right superior frontal gyrus and a positive correlation between the DCDQ-FC General Coordination score and GMV in the left inferior parietal cortex.

**Table 3:** Correlation between DCDQ-FC scores and regional grey matter volume.

**Contrast**	**Correlation**	**Hemisphere**	**Anatomical region**	**MNI coordinates**			***k***	***p***
				**x**	**y**	**z**		
**ASD**								
General coordination	Negative	R	Medial frontal gyrus	6	27	46	101	0.003*
Total	Negative	R	Post-central gyrus	26	−46	58	6	0.042**
**ADHD**								
Total	Negative	R	Superior frontal gyrus	18	68	24	40	0.030**
**TD**								
General coordination	Negative	L	Pre-central gyrus	−45	−8	50	288	0.004*
General coordination	Negative	L	Post-central gyrus	−46	−8	50	5	0.011**
Fine motor/handwriting	Positive	R	Medial frontal gyrus	2	57	3	4	0.045**
**All participants**								
Control during movement	Negative	R	Superior frontal gyrus	22	14	66	192	0.011**
General coordination	Positive	L	Inferior parietal cortex	−36	−39	46	69	0.043**

*ADHD, Attention-Deficit/Hyperactivity Disorder; ASD, Autism Spectrum Disorder; TD, typically developing; k, cluster size; p: p-value*.

* *Significant at p^SVC−FWE^ < 0.004 (Bonferroni corrected for the number of regions of interest)*.

** *Significant at p^SVC−FWE^ < 0.05*.

Importantly, the negative correlation between the DCDQ-FC General Coordination score and GMV in the right medial frontal gyrus within the ASD group at *p*^SVC−FWE^ < 0.05 (*p*^SVC−FWE^ = 0.030) was still a significant when entering ADHD severity as a covariate in the ASD group contrast. However, the correlation between the DCDQ-FC Total score and GMV in the right post-central gyrus did not survived significance threshold within the ASD group.

## Discussion

This study contributes to the investigation of motor impairments in ASD and ADHD. Children with ASD and children with ADHD exhibited impaired motor abilities in all the DCDQ-derived scores compared to TD children. Additionally, most children with ASD or ADHD had an indication or suspicion of DCD. Within the ASD group, coordination abilities were negatively associated with the volume of the right medial frontal gyrus, and within the ADHD group, the total DCDQ score was negatively associated with the volume of the right superior frontal gyrus. Across all participants, control during movement abilities were associated with GMV in the right superior frontal gyrus and coordination abilities were associated with GMV in the left inferior parietal cortex.

Our results showed that an indication or suspicion of DCD was present in the majority of children with ASD and ADHD. Children with ASD exhibited lower scores in all the domains assessed by DCDQ-FC (general coordination, fine motor/handwriting and control during movement) when compared to TD children, hence evidencing an extensive motor impairment in ASD. Additionally, 81.25% of the participants with ASD had an indication of, or suspicion for DCD, similarly to Bhat (12). In a sample of 11,814 children with ASD, Bhat (12) found that 86.9% of the participants were identified as at risk for DCD based on the DCDQ. Recent studies suggested that motor impairments should be taken into consideration in the diagnostic criteria of ASD (5, 12) and our results support that motor impairments are a central feature of ASD. On the other hand, children with ADHD also exhibited motor impairments in all the motor domains assessed (general coordination, fine motor/handwriting and control during movement) when compared to TD children. 72.73% of the participants with ADHD had an indication of, or suspicion for DCD. This proportion is higher compared to previous studies (10). Fliers et al. (10) found that around 30% of girls and 33% of boys in a group of 486 children with ADHD had DCDQ scores below the 10th percentile cut-off, indicating the presence of motor coordination problems. Inconsistencies between studies could be explained by the different age ranges of the participants, the different DCDQ version used, or the fact that Fliers et al. (10) did not include medication intake as an exclusion criterion. Importantly, in our study, none of the children with ASD and ADHD held a formal diagnosis of DCD, which emphasises the fact that motor impairments are under-recognised in these populations. Additionally, the ASD and the ADHD groups did not significantly differ in any of the DCDQ scores, suggesting that ASD and ADHD are similarly affected in terms of the motor abilities assessed by the DCDQ and that the DCDQ does not contribute to differentiate between these disorders.

At the cerebral level, increased impairments in motor coordination were associated with increased GMV in the right medial frontal gyrus within the ASD group. The medial frontal cortex encompasses cortical areas within the median wall of the frontal lobe and has been associated with a wide range of functions including performance monitoring, action anticipation, response inhibition, egocentric spatial processing, and motor coordination (31–33). Medial frontal gyrus volumetric abnormalities have been evidenced in children with DCD compared to TD children (20). Moreover, volume reductions in the medial frontal cortex have been evidenced in patients with ASD in previous studies (34). Overall, it seems that movement coordination abnormalities in ASD are associated with volumetric anomalies in the medial frontal gyrus.

On the other hand, increased motor impairments were associated with increased GMV in the right superior frontal gyrus within the ADHD group. The superior frontal gyrus is implicated in a variety of tasks including motor movement (35–37). In an fMRI study, Licari et al. (36) found decreased activation in the left superior frontal gyrus on a finger-sequencing task in children with DCD. Additionally, the superior frontal gyrus has also been associated with the proactive control of impulsive responses. In a group of healthy adults, Hu et al. (37) found that activation of the right superior frontal gyrus was associated with more efficient response inhibition and less motor urgency. ADHD is associated with motor inhibition deficits (38, 39) and motor impairments in ADHD are characterised by fidgeting and restlessness, possibly explaining the association between motor impairments and GMV in the superior frontal gyrus found in our study.

Our findings should be interpreted in light of some limitations. Poor quality scans were excluded from our sample, decreasing the presence of artefacts but also reducing the size of our sample. Additionally, there was a high percentage of children in the ASD group who presented an ADHD comorbidity, consistent with recent comorbidity reports (3), but potentially influencing our findings. However, this was taken into consideration in our analysis by including ADHD severity in our model. A strength of the present study is that all children with ADHD were non-comorbid and medication naïve and only three participants with ASD had a history of/or were currently taking medication.

Future work should continue exploring the association between volumetric abnormalities and motor impairments in a larger sample of children with non-comorbid ASD, non-comorbid ADHD and children with both ASD and ADHD. Additionally, although the DCDQ is considered a reliable screening tool for detecting children who are at risk for DCD, recent evidence suggests that combining different sources of information when exploring motor abilities in children may be more informative. In particular, using tools such as the Movement Assessment Battery for Children M-ABC (40, 41) may improve the detection of DCD. Future studies should be encouraged to combine DCDQ and MABC measures in order to perform a more exhaustive assessment of potential motor difficulties in children with ASD and ADHD.

In conclusion, this study contributes to the understanding of motor anomalies in ASD and ADHD, as assessed by the DCDQ. Our results evidence frequent motor impairments and a high risk for DCD in populations with ASD and ADHD, underlying the importance of routinely checking for their presence in patients with ASD or ADHD, as well as considering appropriate motor interventions including physical therapy in their treatment. Additionally, the results of this study showed that motor impairments in these populations are associated with volumetric abnormalities in different regions of the brain and contributes to understanding the pathophysiology of motor impairments in these disorders.

## Data Availability Statement

The raw data supporting the conclusions of this article will be made available by the authors, without undue reservation.

## Ethics Statement

The studies involving human participants were reviewed and approved by Ethics Committee of the ULB-Erasme University Hospital in Belgium (P2014/120; CCB: B406201420437). Written informed consent to participate in this study was provided by the participants' legal guardian/next of kin.

## Author Contributions

AS participated in the conceptualization, data acquisition and analysis, and the writing of the article. TV participated in the conceptualization, data analysis, and writing of the article. PV, SB, PD, VD, GP, HR, LV, EW, and PP participated in the conceptualization, data acquisition, and the writing of the article. MS participated in the conceptualization and the writing of the article. ND participated in the conceptualization, data acquisition, supervision, and the writing of the article. IM participated in the conceptualization, data acquisition and analysis, supervision, project administration, funding acquisition, and writing of the article.

## Conflict of Interest

The authors declare that the research was conducted in the absence of any commercial or financial relationships that could be construed as a potential conflict of interest.
