# Supplementary material for: Motor Abnormalities in Attention-Deficit/Hyperactivity Disorder and Autism Spectrum Disorder Are Associated With Regional Grey Matter Volumes
Source: Front Neurol. 2021 May 4;12:666980. doi: 10.3389/fneur.2021.666980 (PMC8129495; doi:10.3389/fneur.2021.666980)
Supplement: Supplementary file 1 [file Table_1.DOCX]

Supplementary material

Supplementary material Table S1. Demographic data of participants included in the analysis, fractionating the ASD group in terms of the presence or absence of ADHD comorbidity.

|  | ADHD (n=22) | | ASD^-^ (n=5) | | ASD^+^ (n=11) | | TD (n=17) | | Between-group difference | | | Post hoc |
| --- | --- | --- | --- | --- | --- | --- | --- | --- | --- | --- | --- | --- |
|  | *M/F* | | *M/F* | | *M/F* | | *M/F* | | *χ2* | *df* | *p* |  |
| Gender | 12/6 | | 4/1 | | 10/1 | | 12/5 | | 1.80 | 3 | 0.61 | ns |
|  | *M* | *SD* | *M* | *SD* | *M* | *SD* | *M* | *SD* | *H* | *df* | *p* |  |
| Age (months) | 122 | 18.51 | 130.2 | 16.54 | 125.55 | 16.77 | 126.12 | 19.48 | 1.18 | 3 | 0.76 | ns |
| IQ | 100.23 | 13.74 | 96.2 | 20.39 | 101.18 | 17.15 | 118.59 | 14.93 | 12.56 | 3 | 0.006 | ADHD<TD |
| ADHD RS-IV Total Score | 33.91 | 9.96 | 11.4 | 4.04 | 27.45 | 5.35 | 7.35 | 4.8 | 40.41 | 3 | <0.001 | ADHD> ASD^-^,TD/  ASD^+^>TD |
| ADHD RS-IV Inattention | 19.27 | 4.38 | 5 | 2.45 | 17.55 | 4.48 | 4.18 | 2.67 | 38.77 | 3 | <0.001 | ADHD> ASD^-^,TD/  ASD^+^>ASD-,TD |
| ADHD RS-IV Hyperactivity | 14.64 | 7.32 | 6.4 | 3.44 | 9.91 | 3.81 | 3.18 | 3.4 | 26.54 | 3 | <0.001 | ADHD, ASD^+^>TD |

ADHD: Attention-deficit/hyperactivity disorder; ASD: Autism spectrum disorder; ASD^-^: ASD without comorbid ADHD; ASD^+^: ASD with comorbid ADHD; TDC: typically developing children; M/F: Male/Female; *χ2*: Pearson's Chi-squared test; M: mean; SD: Standard deviation; H: test statistic for the Kruskal-Wallis test; df: degrees of freedom; p: p-value; ADHD RS-IV: ADHD Rating Scale-IV.

Supplementary material Table S2. DCDQ-FC results and between-group differences, fractionating the ASD group in terms of the presence or absence of ADHD comorbidity.

|  | ADHD (n=22) | | ASD^-^(n=5) | | ASD^+^ (n=11) | | TD (n=17) | | Between-group difference | | | Post hoc |
| --- | --- | --- | --- | --- | --- | --- | --- | --- | --- | --- | --- | --- |
|  | *M* | *SD* | *M* | *SD* | *M* | *SD* | *M* | *SD* | *H* | *df* | *p* |  |
| Total | 51.18 | 12.31 | 51.4 | 6.56 | 38.45 | 13.57 | 67.76 | 4.56 | 29.11 | 3 | <0.001 | ADHD, ASD^+^<TD |
| Control During Movement | 22.09 | 6.11 | 17 | 3.54 | 17.27 | 6.53 | 27.18 | 2.3 | 21.06 | 3 | <0.001 | ASD^-^, ASD^+^<TD |
| Fine Motor/Handwriting | 12 | 4.4 | 16.4 | 1.82 | 9.91 | 4.01 | 17.82 | 2.32 | 23.85 | 3 | <0.001 | ADHD, ASD^+^<TD |
| General Coordination | 16.64 | 4.58 | 18 | 2.45 | 11 | 4.84 | 22.76 | 1.71 | 30.16 | 3 | <0.001 | ADHD, ASD^+^<TD |

ADHD: Attention-deficit/hyperactivity disorder; ASD: Autism spectrum disorder; ASD^-^: ASD without comorbid ADHD; ASD^+^: ASD with comorbid ADHD; TDC: typically developing children; M: mean; SD: Standard deviation; F: test statistic for ANOVA; df: degrees of freedom; p: p-value.
